# One-pot synthesis of corolla-shaped gold nanostructures with (110) planes†

**DOI:** 10.1039/d0ra00715c

**Published:** 2020-02-26

**Authors:** Xiaochuan Xu, Haipeng Zhang, Bin Liu, Jianhui Yang

**Affiliations:** Key Laboratory of Synthetic and Natural Functional Molecule Chemistry (Ministry of Education), Shaanxi Key Laboratory of Physico-Inorganic Chemistry, College of Chemistry & Materials Science, Northwest University Xi'an 710069 P. R. China jianhui@nwu.edu.cn; Xi'an Changfeng Research Institute of Mechanism and Electricity Xi'an 710065 P. R. China

## Abstract

In this work, corolla-shaped gold nanostructures with (110) planes were successfully synthesized using ethylenediaminetetraacetic acid (EDTA) and polyvinylpyrrolidone (PVP) as the co-reductants and shape-directing agents. The structure and the mechanism of the nanostructures were investigated by scanning electron microscopy (SEM), transmission electron microscopy (TEM), X-ray diffraction (XRD), X-ray photoelectron spectroscopy (XPS) and electrochemical characterization. On the basis that surface energies of different gold crystallographic planes are in the order *γ*(111) < *γ*(100) < *γ*(110), the (110) plane has the highest surface energy among the low-index planes. The gold nanocrystals with exposed high-energy planes are important in facilitating their potential applications such as highly efficient catalysts. This research is of great significance for the subsequent work on the synthesis of nanocrystals dominated by high-energy crystal planes.

## Abstract 2

The corolla-shaped gold nanostructures with (110) planes were synthesized using ethylenediaminetetraacetic acid and polyvinylpyrrolidone as the co-reductants and shape-directing agents.

## Graphical abstract

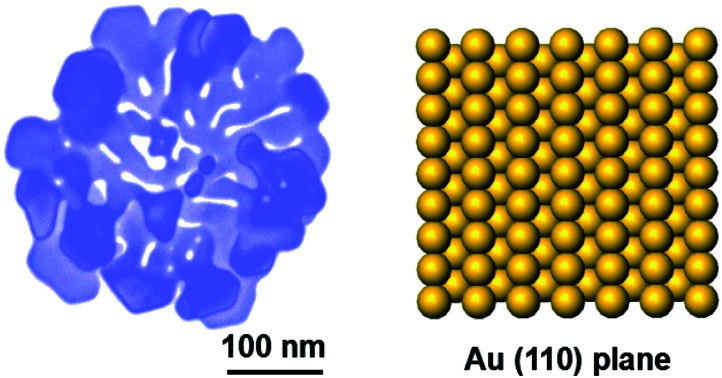

## Introduction

Two-dimensional (2D) nanomaterials have exhibited superior chemical/physical properties, having great promise for various applications such as biosensors,^1,2^ surface-enhanced Raman scattering (SERS),^3^ and catalysis.^4^ Since the discovery of graphene by Andre Geim and Konstantin Novoselov in 2004,^5^ a variety of 2D materials including graphene have been studied. To date, 2D nanomaterials (such as MoS_2_, MnO_2_) and their nanocomposites have been studied and implemented in a variety of applications.^6,7^ Gradually researchers have made some explorations on 2D nano-gold materials (such as gold nano-sheets, gold nano-prisms), especially gold nano-sheets, which show excellent physical and chemical properties.^8,9^

During the past years, we have witnessed rapid advances of using 2D nanomaterials for various applications.^10,11^ In this work, we mainly focus on recent progress of 2D gold nanomaterials. Gold nanosheets with various morphologies have been reported, but almost all of the gold nanosheets that have been reported are mainly (111) crystal planes.^12–14^ The synthesis of gold nanosheets with (111) crystal planes has been relatively mature. However, morphology-controlled synthesis of 2D gold nanomaterials dominated by (110) crystal planes with additional organic-terminated ligands has not been reported. On the basis of the planes that surface energies of different gold crystallographic planes are in the order *γ*(111) < *γ*(100) < *γ*(110),^15^ the (110) planes has the highest surface energy among the low-index planes. It is widely accepted that the synthesis of gold nanocrystals with exposed high-energy planes is an important and challenging work because these planes can endow nanocrystals with a high activity, thus facilitating their potential applications such as highly efficient catalysts.

Herein we propose a one-pot aqueous method for synthesizing 2D gold nanostructures dominated by (110) planes with ethylenediaminetetraacetic acid (EDTA) and polyvinylpyrrolidone (PVP) together as a reductants and shape-directing agents. Observably, these gold nanostructures are basically corolla-like by SEM and TEM investigation. The mechanism of the nanostructures formation further was revealed by XRD and XPS. Electrochemical characterization shows different surface properties of (110) and (111) plane, further confirming that our synthesized gold nanostructure is different from the previous gold nanosheets dominated by (111).

## Results and discussion

**Fig. 1:**
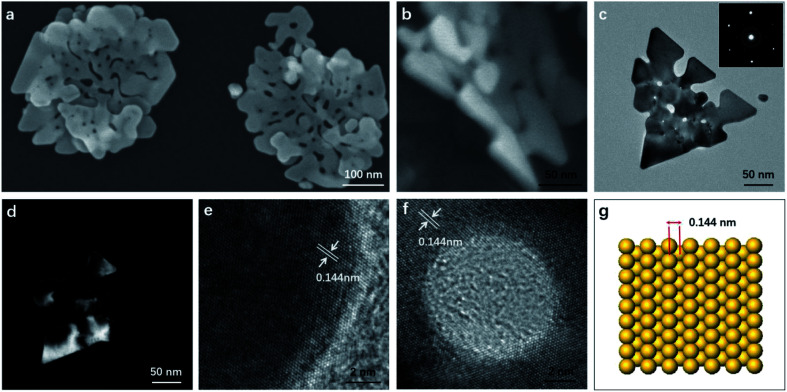
(a and b) SEM, (c) bright-field TEM and (d) dark-field TEM images of the corolla-shaped gold nanostructures. The inset in (a) shows the thickness of a corolla-shaped gold nanostructure. The inset in (c) is the corresponding SAED pattern. (e and f) High-resolution TEM images taken from the corner and the area around a hole of the corolla-shaped gold nanostructure in (c), respectively. (g) The (110) orientation crystal plane simulation diagram.

The as-synthesized products were characterized by SEM and TEM. Fig. 1a shows the typical SEM image of the gold nanostructures. Interestingly, the synthesized product showed a nanostructure similar to the corolla. They show a more complex but generally consistent pattern, which has rarely been previously reported for gold nanostructured materials. The thickness of the nanostructures is determined by measuring a particle standing against the substrate (Fig. 1b). The size and thickness of corolla-shaped Au nanostructures are about 300 nm and 15 nm, respectively. The corolla-shaped nature of the gold nanostructure is further confirmed by the TEM image as shown in Fig. 1c. The inset in Fig. 1c shows the corresponding selected area electron diffraction (SAED) pattern of a corolla-shaped gold nanostructure. The diffraction spots with a six-fold rotational symmetry could be indexed to (220) planes of face-centered cubic (fcc) gold.^16^ The dark-field electron microscopy technique has been proved to be a mature method to observe defects on metal nanoparticles as faces linked by defects may appear with different contrasts. These defects are formed as a result of a minimization of the twin boundary energy.^17^Fig. 1d shows the corresponding dark-field TEM image of corolla-shaped gold nanostructures in Fig. 1c. Alternate dark and light regions were observed on the corolla-shaped gold nanostructures, indicating that there is a height difference in the vertical direction of the corolla-shaped gold nanostructures or the surface of these nanostructures is uneven, which consistent with the observation of SEM and TEM (Fig. 1a and b), due to they are very thin and may be bending.^16^Fig. 1e and f present the high-resolution TEM of corolla-shape gold nanostructures at the edge and around a hole, respectively. The inter-plane distances of 0.144 nm were measured from the high-resolution TEM images for all lattice fringes, which is indexed as (220) planes of metallic gold. These observations indicate that the top and bottom surfaces of the corolla-shaped gold nanostructures are dominated by (110) planes. The typical simulation diagram of gold (110) is shown in Fig. 1g, the arrangement of the surface lattice of the two crystal planes can be seen intuitively in the crystal plane simulation diagram. It can be known from the high-resolution TEM and SAED patterns that the upper and lower surfaces of corolla-shape gold nanostructures have a single crystal structure which dominated by (110) orientation plane. Fig. S1† shows the UV-vis absorption spectrum with a 540 nm peak and a broad absorption peak between 800 to 900 nm for the in-plane and out-of-plane polarizations assigned to the dipole and dipole and higher reorder multipole plasmon resonances of the corolla-shaped gold nanostructures in solution.

**Fig. 2:**
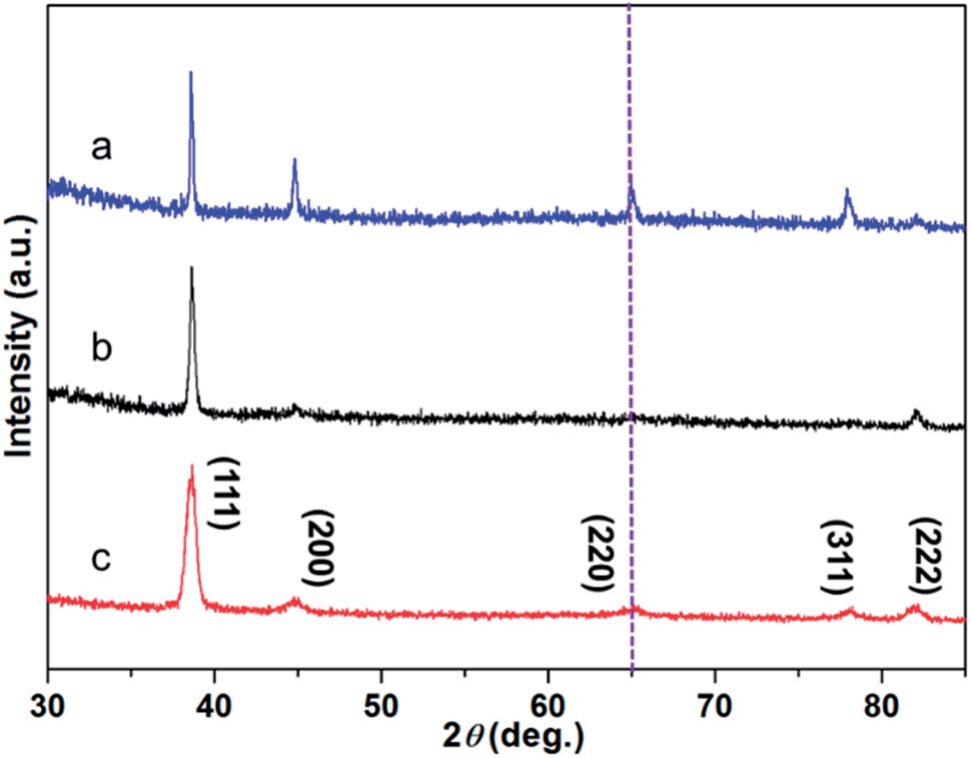
XRD pattern of gold nanostructures synthesized by (a) EDTA, (b) PVP, (c) EDTA and PVP, respectively.

EDTA is one of the polyamino carboxylic acids, often used as a chelating agent. In addition, it can also be used as reducing agent and shape-directing agents for the synthesis of gold nanostructures.^18^ It can be seen from Fig. 2a that the gold nanostructures synthesized with EDTA have the five typical diffraction peaks of gold nanostructures which belong to (111), (200), (220), (311), and (222) diffraction surfaces, respectively (JCPDS card no. 04-0784). The diffraction peaks are relatively strong because the gold nanostructures grow along different crystal planes when synthesized with EDTA (Fig. S2a†).^18^ PVP was often used to prepare metal nanoparticles, in which the polyvinyl skeleton with strong polar pyrrole rings interact directly with the particles surface and strongly influence particles shape.^19–22^ The gold nanostructures synthesized by PVP alone as a reducing agent and stabilizer are mainly (111) crystal planes (Fig. S2b and c†).^19^ XRD pattern also revealed the crystalline nature of the gold nanostructures synthesized by PVP dominated by (111) crystal planes, only the diffraction peaks of the (111) and (222) crystal planes can be seen in the Fig. 2b, indicating that the nanosheets are dominated by the (111) plane (Fig. S2b and c†). Notably, compared to gold nanosheets synthesized with PVP, when EDTA and PVP were used as co-reducing agents and shape directing agents, five well-resolved diffraction peaks of corolla-shaped gold nanostructures were observed in the Fig. 2c. In particular, the diffraction peak at 64.65° belongs to the main crystal plane of the corolla gold nanostructures, which is attributed to the (220) diffraction surface of the face-centered cubic metal gold.

**Fig. 3:**
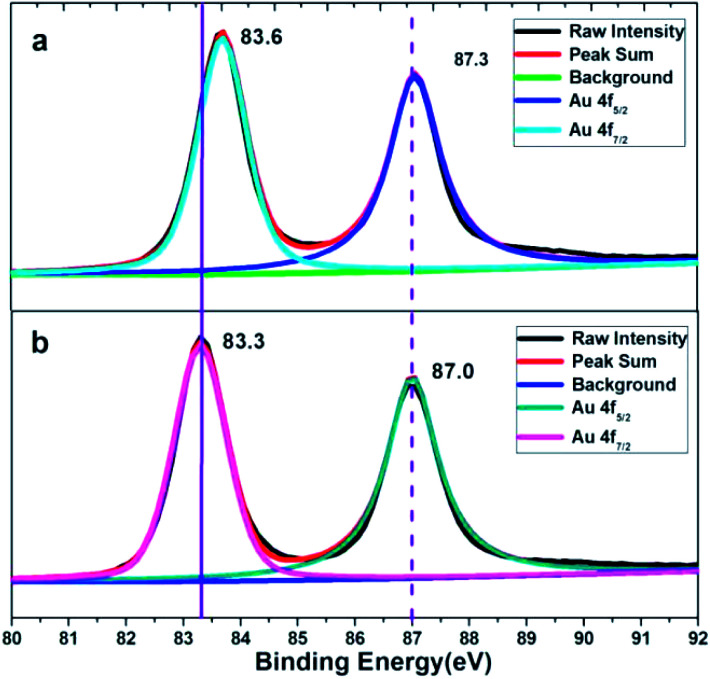
XPS pattern of gold nanostructures synthesized by (a) PVP, (b) EDTA and PVP, respectively.

XPS characterization of the obtained corolla gold nanostructures were further performed to analyze the role of PVP and EDTA in influencing the (110) crystal plane growth. Fig. 3 shows the XPS spectra of Au 4f spectra of gold nanostructures synthesized using EDTA and PVP as reducing agents and coating agents. For comparison, gold nanostructures were synthesized using PVP as functional reagents. A significant negative shift of the binding energy for the Au 4f of the corolla gold nanostructures compared with those of the bulk Au (from 87.7 and 84.0 eV for 4f_5/2_ and 4f_7/2_, respectively, to 87.0 and 83.3 eV).^23,24^ When EDTA and PVP coexist, the product, corolla gold nanostructures, has greater negative shift of the binding energy for the Au 4f. The shift in the binding energy of the Au 4f has frequently been attributed to several factors including material valence, size, geometry or morphology which is connected with the crystallographic orientation (different structural arrangement in the surface atoms) of the gold nanostructures.^25^ Moreover, a negative shift of the binding energy was also attributed to a non-homogeneous charging between the gold nanostructures and its supporting material.^26^ According to the image shown in Fig. 1a and b, the size of gold nanostructures exceeds the 300 nm, and the size of corolla gold nanostructures is similar to that of gold nanosheets synthesized with PVP as a reducing agent. Therefore, one might rule out the size effect and attribute the observed negative shift of the Au 4f binding energy to the geometric/morphology effect. Because the surface atom density of the gold nanostructures dominated by (110) crystal plane is lower than that of the (111) crystal plane,^27^ compared with the coordination number of the pyrrole rings of PVP and the surface of the (111) crystal plane, the carboxyl group of EDTA and the pyrrole rings of PVP have less coordination with the surface of the nanocrystal (110) crystal plane, resulting in a larger negative shift in binding energy.^28–31^ we speculate that different negative shifts are most likely caused by different crystallographic orientation of gold nanostructures.

**Fig. 4:**
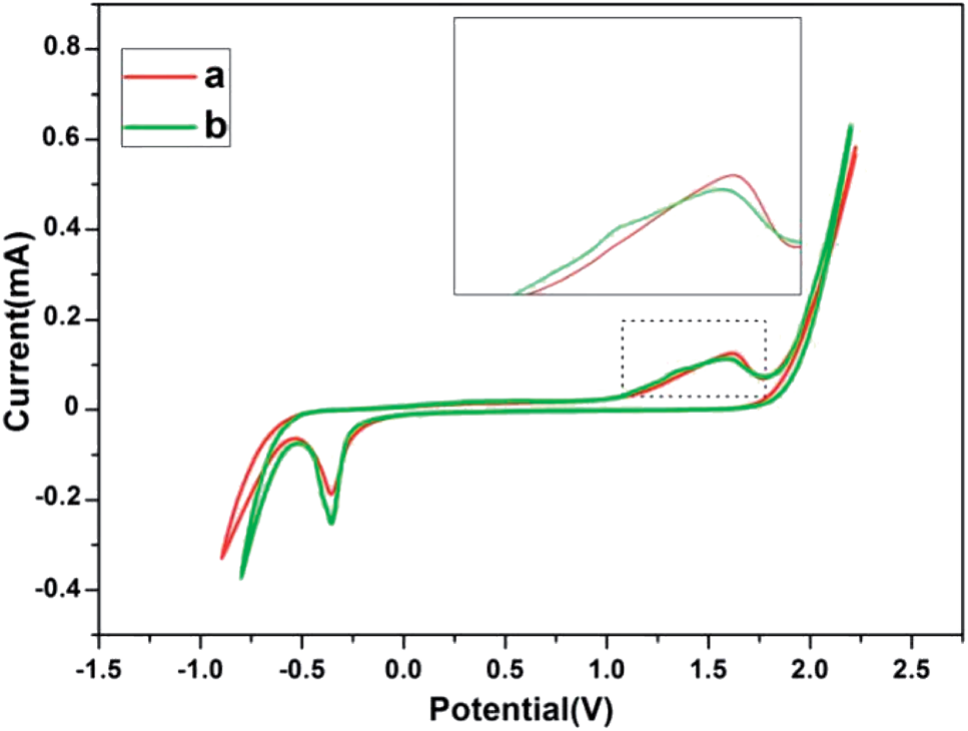
CVs of (a) gold nanosheets and (b) corolla-shaped gold nanostructures.

The electrochemical behavior of a gold electrode is strongly influenced by the superficial structure of the electrode surfaces.^32^ According to the previous reports, gold nanocrystals composed of different (110) and (111) crystal planes have different electrochemical performances.^33,34^ The electrochemical behavior of gold nanosheets dominated by (111) crystal planes synthesized by PVP, and that of corolla gold nanostructures dominated by (110) crystal planes were compared and showing different characteristics in the Au oxide formation and dissolution region. The cyclic voltammograms (CVs) of Au (110) and (111) single crystal electrodes in 0.01 M H_2_SO_4_ give a reliable reference.^32,34^ For Au (111), the main oxidation peak about at +1.50 V. However, for Au (110), one see a main peak at +1.33 V and a broad and shoulder peak at about +1.50 V. The corolla-shaped gold nanostructures surface was characterized by electrochemical methods for further verification. Fig. 4 gives specific information that the CVs of gold nanosheets and corolla-shaped gold nanostructures in 0.01 M H_2_SO_4_. Enlarged CVs in Fig. 4 can observe the difference between two different crystal planes (110) and (111) more intuitively for the positive scan in the region of 1.0–1.75 V for gold nanostructures. The result peaks indicated here can be assigned with respect to results compared with single crystalline electrode.^33,35,36^ Although the characteristic peak of (110) crystal planes in CVs curves is not stronger than that of (111) crystal planes, we still can deduce the corolla-shaped gold nanostructures with (110) planes combined the results of TEM and XRD.

PVP could improve the monodispersity of corolla-shaped gold nanostructures and conferred higher colloidal stability of functionalized corolla-shaped gold nanostructures which may enable potential applications. These gold nanostructures are coated and stabilized with EDTA and PVP. We can clearly observe the coating layers in the TEM image (Fig. S3†) even the sample was washed three times before the TEM investigation. EDTA also acts as a bifunctional reagent, while reducing HAuCl_4_, it also serves as a particle capping agent.^18,37^ EDTA could be oxidized through decarboxylation by highly oxidative chemicals.^38^ HAuCl_4_ is a powerful oxidant with high reduction potential (+0.99 V *vs.* standard hydrogen electrode, SHE) and EDTA serves as an electron donor for HAuCl_4_.^39–41^ According to the crystal growth theory (LaMer mechanism),^42,43^ the surface free energy of the fcc crystal is on the order of *γ*(110) > *γ*(100) > *γ*(111). The shape of fcc metallic nanostructures is mainly determined by the competitive growth of crystal planes with different orientations. In the previous report,^37^ the presence of stabilizing agent, such as CTAB, can affect the growth of gold nanoparticles using EDTA as the reducing agent. The perpendicular growth on the (111) plane is inhibited and the parallel growth along the (110) or (100) direction is enhanced. It results in the formation of anisotropic Au nanocrystals with (111) planes. EDTA contains four carboxylic acid groups (–COOH), polymer stabilizers PVP contains an N–C=O group of pyrrole rings, both of them have the function of suppresses the growth speed of specific crystal facets.^44^ In the current synthesis, PVP plays a dual role as reducing agent and stabilizing agent for the formation of gold nanosheets with (111) planes (Fig. S2b and c†). The corolla-shaped gold nanostructures are produced *via* the redox reaction among AuCl^4−^, EDTA and PVP, which work together to promote formation of corolla-shaped gold nanostructures with the (110) surface, simultaneously, the by-product of Cl^−^ is also present, oxidative etching of gold atoms by Cl^−^ ions leads to the formation of crevasses.^45^ The concurrent crystal growth and oxidative etching on the surface of nanocrystals promote the formation of corolla-shaped gold nanostructures.

## Experimental methods

### Materials

Chloroauric acid tetrahydrate (HAuCl_4_·4H_2_O, 99.9%), ethylenediaminetetraacetic acid (EDTA), polyvinylpyrrolidone (PVP, MW = 10 000), in all experiments, we used ultrapure water (Milli-Q, 18.2 MΩ cm), which was prepared by using a Millipore Milli-Q water system. All reagents were purchased from Aladdin and were used directly without further purification. All glassware used in the experiments was cleaned in a bath of freshly prepared aqua regia (HCl : HNO_3_ = 3 : 1) and rinsed thoroughly with ultrapure water.

### Synthesis of corolla-shaped gold nanostructures

In a typical synthesis for corolla-shaped gold nanostructures, 3.4 mg of HAuCl_4_·4H_2_O, 2.9 mg of EDTA and 22.2 mg of PVP dissolved in 100 mL of ultrapure water in a conical flask (the concentration of HAuCl_4_ is 10^−4^ mol L^−1^; the molar ratio of HAuCl_4_, EDTA and PVP is 1 : 1 : 10) was heated to boiling under vigorously stirring and kept for 10 minutes. The suspension was cooled to room temperature and centrifuged at 8000 rpm for 15 min. The precipitates were collected and washed with ultrapure water 3 times, and finally redispersed in 1 mL of ultrapure water.

### Characterization

FE-SEM images for corolla-shaped gold nanostructures were recorded on a Hitachi SU-8010 scanning electron microscopy with an operating voltage of 5 kV. TEM and HRTEM images were performed using a Talos F200X transmission electronic microscope operated at 200 kV. The water suspension of the sample was dropped and dried on Cu grid supported by holey carbon film for TEM measurement, where an equipped with a SAED pattern were used. UV-vis absorption spectrum was obtained using a UV-1800 UV-vis spectrometer. X-ray diffraction patterns were obtained by using a D8 Advance (30 kV, 15 mA). XPS data was collected on a PHI5000 VersaProbeIII. Cyclic voltammetry of corolla-shaped gold nanostructures with (110) crystal plane and gold nanostructures with (111) crystal plane in 0.01 M sulfuric acid solution was performed using a three-electrode system at an electrochemical station (CHI660E). A Pt coil and an Ag/AgCl electrode were used as the counter and a reference electrode, respectively. The respective scan rate and scan range were 50 mV s^−1^ and −1.5–2.2 V.

## Conclusions

We presented a novel strategy for the synthesis of 2D corolla-shaped gold nanostructures with (110) planes. EDTA and PVP together favors the kinetically-controlled production of corolla-shaped gold nanostructures which improves (110) crystal plane yield. Future, we could envision that corolla-shaped gold nanostructures dominated by the (110) crystal plane would be applicable to many useful applications, that based on its higher surface energy than traditional nanoplates dominated by the (111) crystal plane.

## Conflicts of interest

There are no conflicts to declare.

## Supplementary Material

RA-010-D0RA00715C-s001
