# Supplementary material for: One-pot synthesis of corolla-shaped gold nanostructures with (110) planes
Source: RSC Adv. 2020 Feb 26;10(14):8286–90. doi: 10.1039/d0ra00715c (PMC9049912; doi:10.1039/d0ra00715c)
Supplement: RA-010-D0RA00715C-s001 [file RA-010-D0RA00715C-s001.pdf]

Electronic Supplementary Material (ESI) for RSC Advances.
This journal is © The Royal Society of Chemistry 2020

Supplementary information

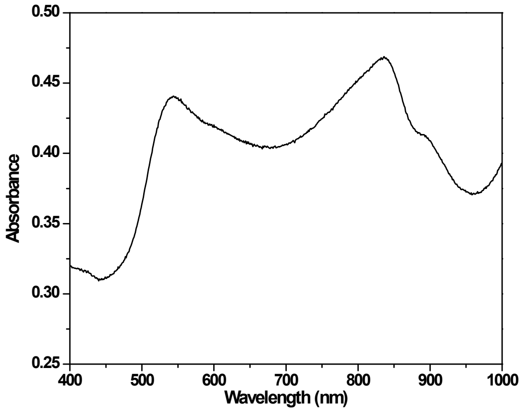

Fig. S1 UV-vis absorption spectrum of the aqueous solution of corolla-shape Au nanostructures.

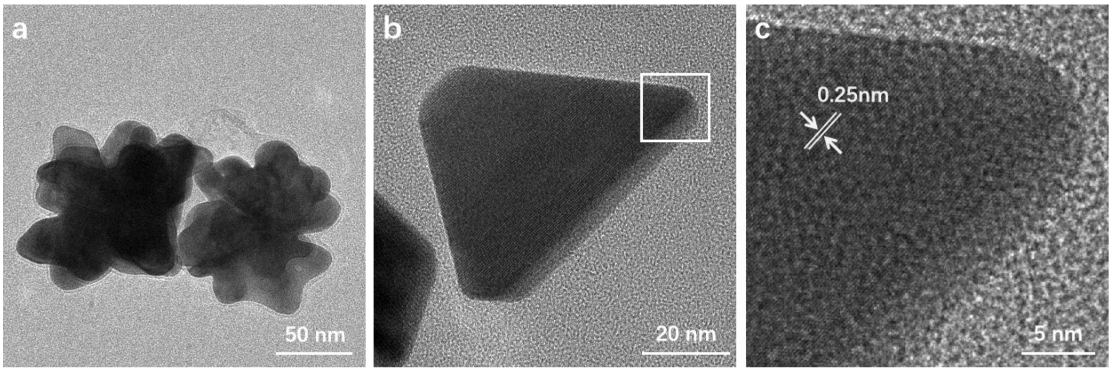

Fig. S2 TEM images of (a) Au nanoflowers and (b) nanosheets synthesized only using EDTA and PVP, respectively. (c) High-resolution TEM image of Au nanosheets taken in (b) using a boxed spot.

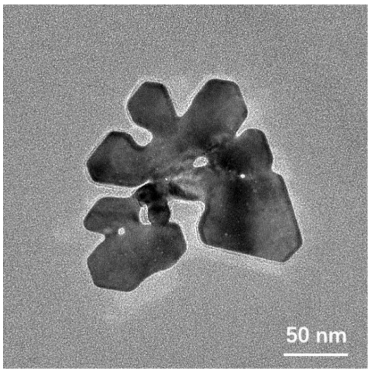

Fig. S3 Additional TEM image of a corolla-shape Au nanostructure.
